# Cellulitis of Neck Accompanied by Œdema Glottidis; Difficult Laryngotomy

**Published:** 1881-01

**Authors:** G. Frank Lydston

**Affiliations:** House Surgeon, Charity Hospital, New York


### ARTICLE VII.

### CELLULITIS OF NECK ACCOMPANIED BY ŒDEMA GLOTTIDIS;" DIFFICULT LARYNGOTOMY. By G. FRANK LYDSTON, M.D., House Surgeon, Charity Hospital, New York.

I submit the following history of a very curious case, which may prove interesting. The patient, J. M. Labover, æt. 27 years, entered hospital November 30, with the following history. He stated that he had been on a spree for a week past, and that he had eaten no food for four days, but that he was perfectly well until three days previous to admission. At that time he noticed a small sore beneath the ramus of the jaw on the left side of neck, which was followed by considerable swelling, and shortly after its appearance patient was seized with a severe chill, followed by fever, headache, thirst and vomiting—the headache being especially marked. There was slight pain at situation of ulceration above mentioned. The headache had grown very severe, especially during last two days, there being exacerbations of pain, followed by nausea and vomiting. This pain is now referred principally to crown of head and occiput. Has suffered from insomnia since beginning of trouble.

On admission, a diffuse, boggy swelling appeared on left side of neck and face, extending to clavicle and somewhat below it, and there being some swelling of right side of the neck. There was no redness, excepting in posterior part of neck on the left side; no heat, and very slight tenderness on pressure. On the left side of neck, just below the ramus of the jaw, was a red, elevated, and somewhat ulcerated oval patch, about one inch in length by half an inch in breadth, this being surrounded by well-marked induration. The lymphatic glands of neck, and parotid and submaxillary glands, were enlarged and tender. No history of injury could be obtained.

An examination of throat revealed considerable swelling and redness of fauces, especially on the left side, in which situation a tumor could be discerned. Reflex sensibility was so exaggerated that the slightest attempt at examination of the throat caused retching. When the first examination was made, patient was able to stand, and being a remarkable specimen of muscular development, measurements of chest and arms were made, the examination not seeming to exhaust him much. A short time after the examination, however, the patient requested permission to go to bed, saying that he was too ill to stand; and a bed was given him at 12 m., Dec. 2d. He lay but a short time, however, when he suddenly sprang out of bed and began staggering about the ward, saying that his head pained him so that he could not lie still.

Respiration appeared to be quite embarrassed, although patient made little complaint. I ordered him to bed, and administered liq. morphiæ, U. S., ʒij. The patient was not at all violent, but refused to assume the dorsal decubitus, apparently on account of dyspnœa, although he referred it to the intense cephalalgia from which he was suffering. A saline cathartic was given, followed by stimulants and quiniæ sulph. gr. v every three hours. The lotio plumbi et opii was applied to the neck. The temperature at 1 p. m. was 103½ Fahr., pulse 120, full, but soft and compressible. Shortly after taking the morphia, patient drank freely of beef tea, and in a short time fell into a heavy slumber, from which he could with difficulty be aroused for the administration of remedies. He remained in this condition until 5:30 p. m., when he aroused somewhat, but would swallow nothing, and resisted the administration of remedies by the rectum. He speedily relapsed into a semi-comatose condition, from which he did not again arouse. The lotio plumbi et opii was now replaced by hot poultices, renewed every fifteen minutes, with stimulants per rectum, and hypodermics of tr. digitalis and ammon. carb. I did not see the patient again until 10 p. m., but during this time, his dyspnœa rapidly increased, and patient had three short clonic convulsions. He was seen by the house staff at 9 p. m., and hypodermics of whisky and digitalis given. The pulse at that time was very feeble and intermittent, and the respiration was markedly obstructed. At 10 p. m. I again saw the patient. Respiration was now greatly embarrassed, the obstruction being chiefly confined to inspiration, the pulse still feebler than when last seen, and very intermittent.

The jaws were tightly set, and it was with great difficulty that I succeeded in introducing my finger into the pharynx, and experienced considerable trouble in reaching the glottis, the larynx being displaced downwards and to the right.

The glottis was found to be very œdematous and the fauces very much swollen. The swelling of the neck was now greatly increased, having extended to a considerable degree across the median line and to the posterior portion of neck, giving the head and face a very peculiar appearance. The eyes were now rolled upwards, and fixed, and pupils dilated, face and lips cyanosed, respiration still more obstructed.

Respiration became completely suspended several times for a short period. At 11 p. m., the pulse and respiration being very feeble, laryngotomy was performed. The swelling and infiltration of tissue was so great that absolutely nothing in the way of landmarks was perceptible on palpation, and as the patient was very stout, and muscular, and the neck very short, the difficulty in reaching the larynx was necessarily very great. An incision was made in the median line, and a careful but rapid dissection made in that situation.

The hæmorrhage was very profuse, as a result of venous engorgement. The larynx was displaced about half an inch to the right, by pressure of inflammatory products.

The pulse became imperceptible, and respiration entirely ceased, necessitating a rapid termination of the operation, and an incision being made in crico-thyroid space, a large tracheotomy tube was introduced, and by artificial respiration, patient restored so that heart's action and respiration were resumed.

Cyanosis now disappeared, but patient still remained comatose.

Stimulants, and digitalis, and shortly afterward, atropia, were given hypodermically. At 1:30 a. m. the temperature was 104° Fahr. Respiration grew very peculiar, there being a series of forcible inspirations, with occasionally complete suspension of movements, followed by several gasping inspirations.

On performance of artificial respiration breathing was resumed as before. After administering the atropia, respirations grew very forcible, and 60 per minute. A sponge bath was given at 2 a. m. The trachea was several times partially cleared of blood, by the use of a large syringe and rubber tubing, but not proving efficient, forcible expulsion of the blood was caused by throwing weight forcibly against the thorax, at termination of a full inspiration, an assistant catching the blood quickly with a sponge. In this manner quite a quantity of blood was removed. The patient remained comatose, and died at 4:30 a. m., five and a half hours after operation, apparently from failure of respiratory sense. On autopsy, nothing distinctive was discovered save œdema glottidis, which was very marked. Absolutely nothing was discovered to account for the cerebral symptoms which were manifested.

### REMARKS.

The points of interest in the case are these: The evident malignity of the case, as evidenced by its rapid course, after admission to the hospital; its obscure nature; the occurrence of intense œdema glottidis as a result of the cellulitis of neck, and apparent presence of an element of a toxical nature. The inference which I drew from the symptoms, and history of the case was, either that he had received an injury while intoxicated, which subsequently became inocculated with some septic poison with cellulitis as a consequence, or that he had been stung by some insect. The case in some respects, was not unlike "malignant pustule" so-called. There was evidently some interference with the respiratory sense, which was not accounted for by any pathological change in the brain or its membranes.

Congestion from obstruction to the return circulation, may possibly have been the cause, but there were no post-mortem evidences of it. I can not imagine a more unfavorable case for laryngotomy unless in such cases as of tumor covering the larynx and trachea. The operation itself was successful inasmuch as it fulfilled the indications present, and if the result of the case had depended entirely upon the œdema glottidis, it would have been favorable. It illustrates very forcibly the fact that no delay in operating is admissible in cases of the kind, and in a similar case, I should operate much earlier. The prompt stimulation of the respiration by atropia, and the method adopted for clearing the trachea of blood, were also interesting features of the case.

---

THE INTERNAL ADMINISTRATION OF THE ETHYL BROMIDE.—Satisfied as to this, we were determined to obtain information as to its action when administered internally; and for that purpose gave to three of the animals respectively 10, 20, and 30 minims of the ethyl. While producing in the larger doses more markedly a slight intoxication, no other serious symptoms arose. Encouraged by this, we commenced taking it ourselves, well diluted, first in doses of 5 and 10, and then 25 and 30 drops, without discovering any noticeable effect, save that of slight sleepiness induced by the larger doses. A nervous headache, existing during these experiments, seems by the ethyl to have been entirely relieved, which however might have been the case had any other bromide been taken instead. That it may prove of decided benefit in nervous irritation and hysteria is readily to be inferred herefrom.

To the taste it is sweet and pleasant, but heating to the mucous surfaces, and it should be well diluted before it is administered.—*L. Wolff.*

